# Quantitative assessment of the associations between XRCC1 polymorphisms and bladder cancer risk

**DOI:** 10.1186/1477-7819-11-58

**Published:** 2013-03-07

**Authors:** Yeqing Mao, Xin Xu, Yiwei Lin, Hong Chen, Jian Wu, Zhenghui Hu, Yi Zhu, Xianglai Xu, Liping Xie

**Affiliations:** 1 Department of Urology, First Affiliated Hospital, School of Medicine, Zhejiang University, Qingchun Road 79, Hangzhou, Zhejiang Province, 310003, China

**Keywords:** *XRCC1*, Polymorphism, Bladder cancer, Meta-analysis

## Abstract

**Background:**

The *XRCC1* polymorphisms have been implicated in bladder cancer risk, but individually published studies show inconsistent results. The aim of our study was to clarify the effects of *XRCC1* variants on bladder cancer risk.

**Methods:**

A systematic literature search up to September 13, 2012 was carried out in PubMed, EMBASE and Wanfang databases, and the references of retrieved articles were screened. Crude odds ratios with 95% confidence intervals were used to assess the associations between *XRCC1* Arg194Trp and Arg399Gln polymorphisms and bladder cancer risk. Heterogeneity and publication bias were also evaluated.

**Results:**

A total of 14 and 18 studies were eligible for meta-analyses of Arg194Trp and Arg399Gln, respectively. Regrouping was adopted in accordance with the most probable appropriate genetic models. No obvious heterogeneity between studies was found. For overall bladder cancer, the pooled odds ratios for Arg194Trp and Arg399Gln were 1.69 (95% confidence interval: 1.25 to 2.28; *P* = 0.001) and 1.10 (95% confidence interval: 1.03 to 1.19; *P* = 0.008), respectively. After excluding the studies that were not in Hardy–Weinberg equilibrium, the estimated pooled odds ratio still did not change at all.

**Conclusions:**

The meta-analysis results suggest that *XRCC1* Arg194Trp and Arg399Gln polymorphisms may be associated with elevated bladder cancer risk.

## Background

Bladder cancer is an important health problem worldwide. It is the seventh most common malignancy in men and seventeenth in women [1]. An estimated 386,300 new cases and 150,200 deaths from bladder cancer occurred in 2008 worldwide [2]. However, the mechanism of bladder cancer is not completely clear and is considered to be a multifactorial process. The most established risk factors for bladder cancer include cigarette smoking, occupational exposure to arylamines and schistosomal infection [1]. These exogenous mutagens or carcinogens produce a wide range of DNA lesions, bulky DNA adducts, and DNA strand breaks. Epidemiologic evidence has shown that genetic variants at one or more loci result in reduced DNA repair capacity and an increased cancer risk [3-5].

DNA carrying essential heritable information must remain stable in order to undertake its key physiologic functions, but it is continually vulnerable to many types of endogenous and/or exogenous damage; thus, genetic alterations could accumulate and tumorigenesis may occur because of the damaged DNA. The DNA repair system plays a pivotal role in maintaining the genome integrity and stability through the reversal of DNA damage. If accumulated genetic alterations occurred in corresponding DNA repair genes, their reversal capacity could be damaged, possibly increasing the risk of cancer in carriers [6]. A large number of SNPs in common DNA repair genes have been identified [7] and confirmed to be associated with several sporadic cancers [8,9].

X-ray repair cross-complementing group 1 (*XRCC1*), located on chromosome 19q13.2–13.3, with 33 kb in length, is an important component of base excision repair (BER) [10]. BER consists of a series of consecutive steps from the recognition and excision of a damaged base to the ligation of broken points, which are mainly conducted by *XRCC1*. When damage occurs, *XRCC1* recruited by DNA glycosylases, acts as a platform by regulating and coordinating a whole list of BER proteins and single strand break repair (SSBR) machinery [11,12]. Although there are more than 300 validated SNPs in the *XRCC1* gene reported in the dbSNP database (http://www.ncbi.nlm.nih.gov/SNP), two genetic changes including Arg194Trp on exon 6 (rs1799782 in dbSNP, C/T) and Arg399Gln on exon 10 (rs25487 in dbSNP, G/A) are the most extensively studied. Many previous studies have been conducted to evaluate the associations of *XRCC1* polymorphisms with bladder cancer risk. However, the results of these studies remain inconsistent and contradictory, partially because a single study may be too underpowered to detect a possible small effect of the polymorphism on bladder cancer, especially when the sample size is relatively small. Thus, to clarify the effect of *XRCC1* variants (Arg194Trp and Arg399Gln) on bladder cancer risk, we performed a meta-analysis of all eligible studies.

## Methods

### Publication search

We carried out a systematic literature search in EMBASE, PubMed and Wanfang databases, covering all the papers published from their inception to September 13, 2012, using the following key words: (*XRCC1* or X-ray repair cross-complementation group 1) and (bladder cancer or bladder neoplasm or bladder tumor or urothelial cancer or urinary tract cancer) and (polymorphism or variation or variant or mutation or genotype or gene). There was no language restriction. We evaluated potentially relevant papers by checking their titles and abstracts and all the studies matching the eligible criteria were retrieved. Additional studies were identified by a manual search of the references from retrieved articles and reviews.

### Inclusion criteria

Studies included in the present meta-analysis had to meet all the following criteria: (a) evaluation of the *XRCC1* Arg194Trp and Arg399Gln polymorphisms and the risk of bladder cancer, (b) had a case–control design or nested case–control design, (c) had sufficient data for calculating an odds ratio (OR) with 95% confidence interval (CI). If multiple publications from the same population were available, the most recent or largest study was eligible for inclusion in this meta-analysis.

### Data extraction

Data were extracted independently by two authors using a predefined data collection form, with disagreements being resolved by consensus. For each study, the following information was collected: first author’s last name, publication year, the country in which the study was carried out, ethnicity, numbers of cases and controls, genotyping methods, genotypes, and allele frequency information.

### Quality assessment

The quality of each study was independently appraised by the same two authors using the quality assessment criteria, which were modified on the basis of previously published meta-analysis of molecular association studies [13,14]. The criteria consist of seven parameters of quality: representativeness of the cases, representativeness of the controls, ascertainment of bladder cancers, control selection, genotyping examination, Hardy–Weinberg equilibrium HWE) and total sample size. (The criteria are described in detail in Additional file 1: Table S1). Scores ranged from 0 (worst) to 15 (best). Studies scoring <9 were classified as low quality, and those ≥9 as high quality. Disagreements were resolved by a joint reevaluation of the original article with a third investigator.

### Statistical methods

HWE in cases and controls was examined again in our meta-analysis using the goodness-of-fit test (significant at the 0.05 level). The ORs and their 95% CIs were used to calculate and assess the strength of the association between *XRCC1* Arg194Trp and Arg399Gln polymorphism and the risk of bladder cancer. If there was a statistical heterogeneity among studies, the combined ORs and 95% CIs were estimated by the DerSimonian and Laird method [15] in a random-effect model. Otherwise, the ORs were obtained by the Mantel–Haenszel method [16] in a fixed effect model.

ORs 1, 2, and 3 (OR1, OR2, and OR3) were calculated for the genotypes: 1) TT versus CC, 2) CT versus CC, and 3) TT versus CT for Arg194Trp; and 1) AA versus GG, 2) GA versus GG, and 3) AA versus GA for Arg399Gln, respectively. These pairwise differences were used to determine the most appropriate genetic model. If OR1 = OR3 ≠1 and OR2 = 1, a recessive model is implied. If OR1 = OR2 ≠ 1 and OR3 = 1, a dominant model is suggested. If OR2 =1/OR3 ≠ 1 and OR1 = 1, then a complete overdominant model is indicated. If OR1 > OR2 >1 and OR1 > OR3 >1, or if OR1 < OR2 <1 and OR1 < OR3 < , then a codominant model is suggested.

Homogeneity of ORs across studies was tested by a Chi-square-based Q statistic and the I^2^ score. Heterogeneity was considered significant if the *P*-value was <0.10. The value of I^2^ was used to assess the degree of heterogeneity (I^2^ <25% no heterogeneity; I^2^ = 25% to 50% moderate heterogeneity; I^2^ >50% large or extreme heterogeneity).

Sensitivity analysis was performed in which the meta-analysis estimates were computed after omission of every study in turn. Cumulative meta-analyses of associations for each SNP were also conducted through assortment of studies with publication time.

### Evaluation of publication bias

Publication bias was assessed using Begg’s test (rank correlation method) [17] and Egger’s test (linear regression method) [18]. *P* <0.05 was considered to be representative of a significant statistical publication bias. All of the statistical analyses were performed with STATA 11.0 (StataCorp, College Station, TX), using two-sided *P*-values.

## Results

### Characteristics of all included studies

**Figure 1:**
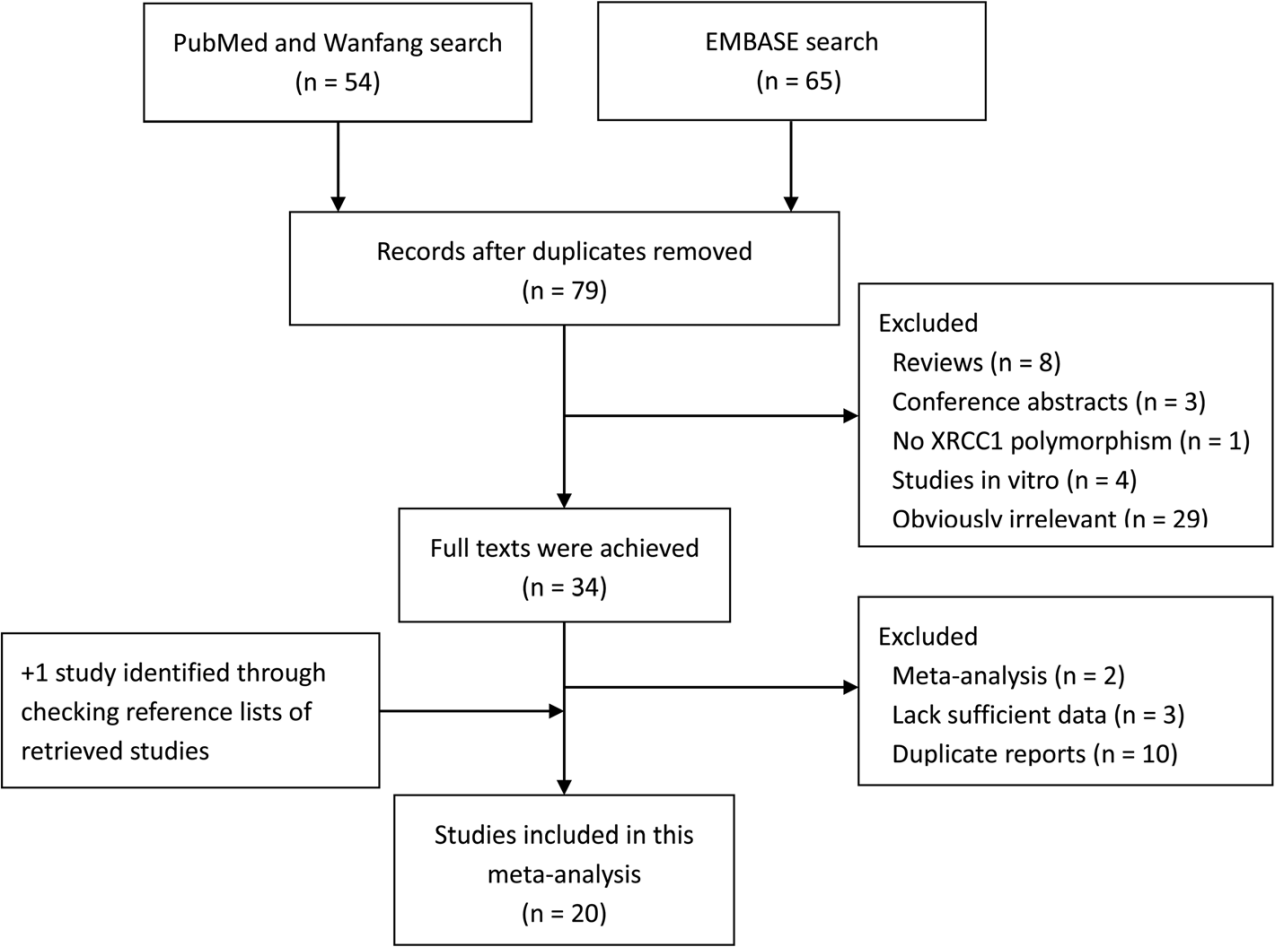
Flowchart of study assessment and selection.

Twenty studies were included in this meta-analysis on the associations of the *XRCC1* genetic polymorphisms with the risk of bladder cancer. Of the selected studies, 14 [19-32] were preliminarily appropriate for meta-analysis of the associations with bladder cancer regarding Arg194Trp, and 18 [19-22,24-28,30-38] were relevant to the association with Arg399Gln. Tables 1 and 2 present the basic characteristics of each study included in our meta-analysis and the corresponding genotype distributions among cases and controls. The literature search and study selection procedures are shown in Figure 1.

**Table 1:** **Main characteristics of the studies included in an analysis of the *XRCC1 *Arg194Trp polymorphism and bladder cancer risk**

**Study**	**Ethnicity**	**Country**	**Sample size (Frequency of T allele, %)**		**HWE in control**	**Quality score**	**Genotyping method**
			**Case no**	**Control no**			
Stern [19]	Caucasian	US	235 (5.63)	213 (8.63)	Yes	8	PCR-RFLP
Wu [20]	Asian	China	155 (33.87)	155 (27.42)	Yes	9	PCR-RFLP
Matullo [21]	Caucasian	Mixed	131 (6.45)	1,094 (6.63)	Yes	13	Taqman
Wu [22]	Caucasian	US	696 (6.43)	629 (6.42)	Yes	11	Taqman
Zhang [23]	Asian	China	242 (33.47)	225 (26.22)	No	12	PCR-RFLP
Sak [24]	Caucasian	UK	547 (5.79)	579 (5.96)	Yes	12	Taqman
Figueroa [25]	Caucasian	Spain	1,150 (6.11)	1,149 (5.72)	Yes	12	Taqman
Andrew [26]	Caucasian	US, Italy	1,029 (6.49)	1,281 (7.15)	Yes	12	Taqman
Hsu [27]	Asian	Taiwan	221 (34.86)	223 (33.26)	No	7	PCR-RFLP
Fontana [28]	Caucasian	France	51 (3.92)	45 (5.56)	Yes	6	Taqman
Narter [29]	Caucasian	Turkey	83 (21.93)	45 (23.61)	Yes	4	PCR-RFLP
Wang [30]	Asian	China	234 (31.62)	253 (23.72)	Yes	8	PCR-RFLP
Bianchino [31]	Caucasian	Italy	32 (12.50)	242 (7.02)	Yes	5	PCR-RFLP
Mittal [32]	Asian	India	212 (10.14)	250 (9.00)	Yes	10	PCR-RFLP

HWE, Hardy–Weinberg equilibrium.

**Table 2:** **Main characteristics of the studies included in an analysis of the *XRCC1 *Arg399Gln polymorphism and bladder cancer risk**

**Study**	**Ethnicity**	**Country**	**Sample size (Frequency of A allele, %)**		**HWE in control**	**Quality score**	**Genotyping method**
			**Case no**	**Control no**			
Stern [19]	Caucasian	US	235 (34.58)	213 (36.55)	Yes	8	PCR-RFLP
Shen [33]	Caucasian	Italy	201 (32.09)	214 (34.11)	Yes	9	PCR-RFLP
Sanyal [34]	Caucasian	Sweden	311 (35.21)	246 (31.71)	Yes	9	PCR-RFLP
Broberg [35]	Caucasian	Sweden	61 (31.97)	155 (28.39)	Yes	9	MALDI-TOF
Wu [20]	Asian	China	155 (27.74)	155 (46.45)	Yes	8	PCR-RFLP
Matullo [21]	Caucasian	Mixed	131 (35.08)	1,094 (33.73)	Yes	11	Taqman
Wu [22]	Caucasian	US	696 (34.01)	629 (33.72)	Yes	10	Taqman
Karahalil [36]	Caucasian	Turkey	100 (32.00)	100 (38.00)	Yes	7	PCR-RFLP
Sak [24]	Caucasian	UK	547 (35.71)	579 (36.52)	Yes	11	Taqman
Figueroa [25]	Caucasian	Spain	1,150 (35.82)	1,149 (33.79)	Yes	11	Taqman
Andrew [26]	Caucasian	US, Italy	1,029 (35.35)	1,281 (36.07)	No	10	Taqman
Hsu [27]	Asian	Taiwan	221 (25.71)	223 (25.46)	Yes	8	PCR-RFLP
Arizono [37]	Asian	Japan	251 (24.30)	251 (26.29)	Yes	8	PCR-RFLP
Fontana [28]	Caucasian	France	51 (34.31)	45 (40.00)	Yes	5	Taqman
Wang [30]	Asian	China	234 (32.26)	253 (29.45)	Yes	7	PCR-RFLP
Bianchino [31]	Caucasian	Italy	32 (43.75)	242 (30.99)	No	4	PCR-RFLP
Mittal [32]	Asian	India	212 (43.40)	250 (37.40)	Yes	9	PCR-RFLP
Zhi [38]	Asian	China	302 (34.93)	311 (29.42)	Yes	10	PCR-RFLP

HWE, Hardy–Weinberg equilibrium.

### Quantitative synthesis

For the Arg194Trp SNP, OR1, OR2, and OR3 were 1.835 (95%CI: 1.343 to 2.507), 1.026 (95%CI: 0.920 to 1.146), and 1.581 (95%CI: 1.154 to 2.165), respectively, suggesting a recessive effect of the putative susceptibility allele T. Thus, the original grouping was collapsed, and CC and CT were combined, in accordance with a recessive model, into a C carrier group, the latter of which was compared with the TT genotype group.

For the Arg399Gln SNP, OR1, OR2, and OR3 were 0.958 (95%CI: 0.850 to 1.080), 1.095 (95%CI: 1.014 to 1.183), and 0.884 (95%CI: 0.785 to 0.997), respectively, indicating that a complete overdominant model was applicable, that is, heterozygotes are at higher risk of bladder cancer than either homozygotes (GG or AA).

**Figure 2:**
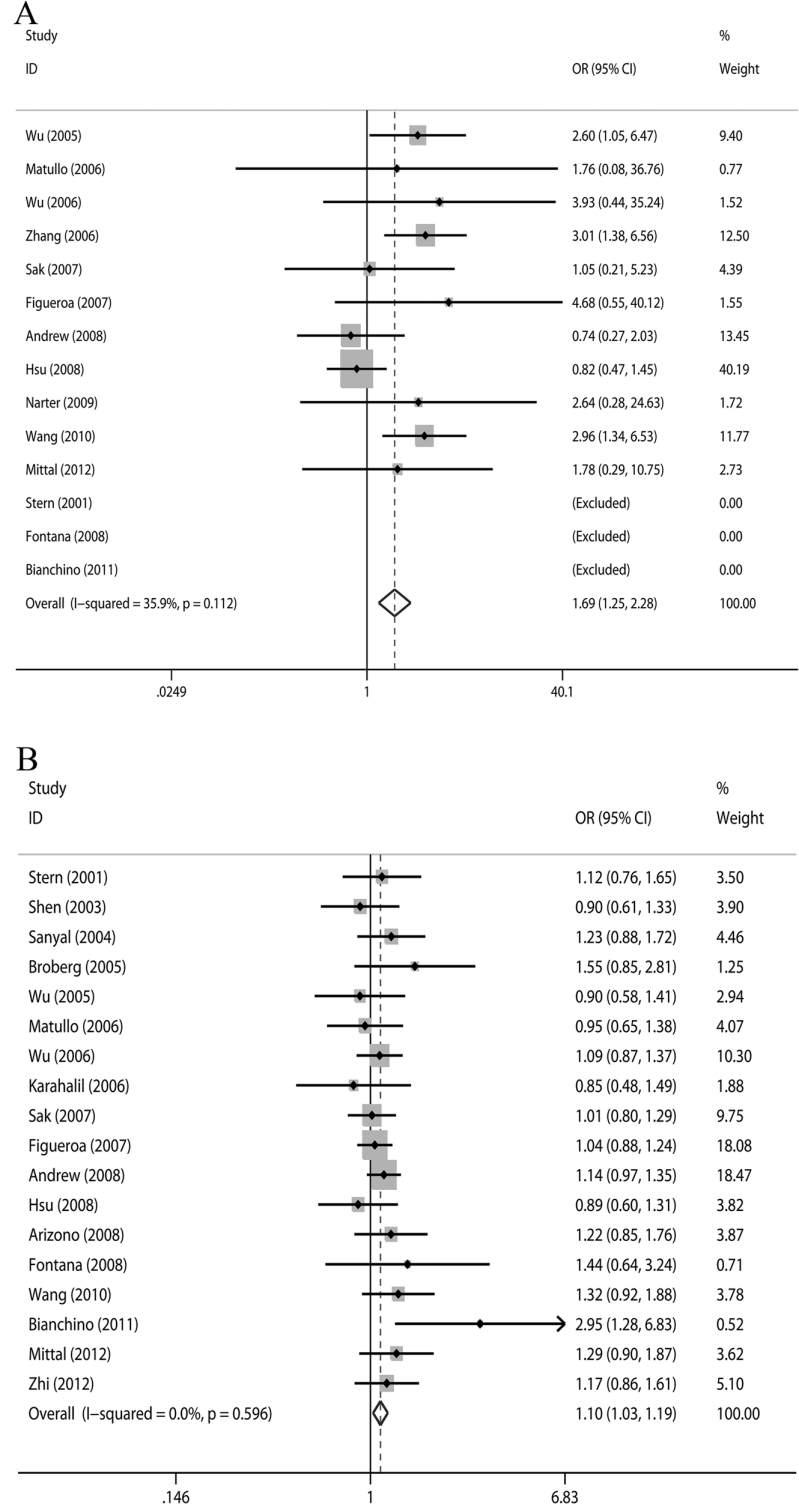
**Odds ratios (ORs) for associations between two single nucleotide polymorphisms (Arg194Trp and Arg399Gln) in the x-ray repair cross-complementing group 1 gene (*XRCC1*) and bladder cancer risk.** The size of the black square corresponding to each study is proportional to the sample size and the center of each square represents the OR. The horizontal line shows the corresponding 95% CI of the OR. Pooled OR is represented by a hollow diamond. **A**) Arg194Trp TT genotypes versus the CC-plus-CT genotype; **B**) Arg399Gln GA genotypes versus the GG-plus-AA genotype. CI, confidence interval.

As shown in Figures 2 and Table 3, the *XRCC1* Arg194Trp polymorphism was associated with an increased risk for bladder cancer in all subjects (OR = 1.69, 95% CI = 1.25 to 2.28, *P* = 0.001). Similarly, the Arg399Gln polymorphism was also found to be significantly associated with increased risk of bladder cancer (OR = 1.10, 95% CI = 1.03 to 1.19, *P* = 0.008).

**Table 3:** **Meta-analysis of the association between the *XRCC1 *Arg194Trp and Arg399Gln genetic polymorphisms and the risk of bladder cancer**

**Polymorphism**	**Stratification factor**	**Sample size**		**Number of studies**	**Test of association**				**Test of heterogeneity**	
		**Case**	**Control**		**OR (95% CI)**	***P*-value**	**z**	**Model**	**I**^**2 **^**(%)**	***P*-value**
Arg194Trp	Overall	4,751	6,102	14	1.69 (1.25-2.28)	0.001	3.39	F	35.9	0.112
	Study in HWE	4,301	5,659	12	2.07 (1.36-3.15)	0.001	3.38	F	0.0	0.575
	Ethnicity									
	Asian	1,051	1,101	5	1.97 (1.04-3.74)	0.038	2.08	R	64.1	0.025
	Caucasian	3,700	5,001	9	1.44 (0.75-2.74)	0.270	1.10	F	0.0	0.538
	Study quality									
	High	3,956	5,111	8	2.08 (1.36-3.18)	0.001	3.36	F	0	0.458
	Low	795	991	6	1.35 (0.88-2.08)	0.352	0.93	R	71.6	0.030
Arg399Gln	Overall	5,654	7,136	18	1.10 (1.03-1.19)	0.008	2.67	F	0.0	0.596
	Study in HWE	4,632	5,641	16	1.08 (1.00-1.18)	0.053	1.94	F	0.0	0.858
	Ethnicity									
	Asian	1,364	1,438	6	1.14 (0.98-1.33)	0.082	1.74	F	0.0	0.562
	Caucasian	4,290	5,698	12	1.09 (1.01-1.19)	0.037	2.09	F	0.0	0.460
	Study quality									
	High	4,407	5,675	10	1.10 (1.01-1.19)	0.028	2.20	F	0.0	0.819
	Low	1,247	1,461	8	1.13 (0.97-1.33)	0.122	1.55	R	28.1	0.204

CI, confidence interval; F, fixed-effect model; HWE, Hardy–Weinberg equilibrium; OR, odds ratio; R, random-effect model.

### Evaluation of heterogeneity

For the Arg399Gln polymorphism, most I^2^ values of heterogeneity were 0% and all *P* values were more than 0.10, indicating no statistically significant heterogeneity between studies (Table 3). Similarly, for the Arg194Trp polymorphism, there was also no obvious heterogeneity between studies.

### Sensitivity analysis

In the sensitivity analysis, the influence of each study on the pooled OR was examined by repeating the meta-analysis while omitting each study, one at a time. This procedure proved that our results were reliable and robust. In addition, when excluding the studies that were not in HWE, the estimated pooled OR still did not change at all (data not shown).

### Cumulative meta-analysis

Cumulative meta-analyses of the two associations were also conducted via the assortment of studies by publication time. The 95% confidence intervals became increasingly narrower with increasing sample size, indicating that the precision of the estimates was progressively boosted by the continual addition of more cases (data not shown).

### Publication bias

**Figure 3:**
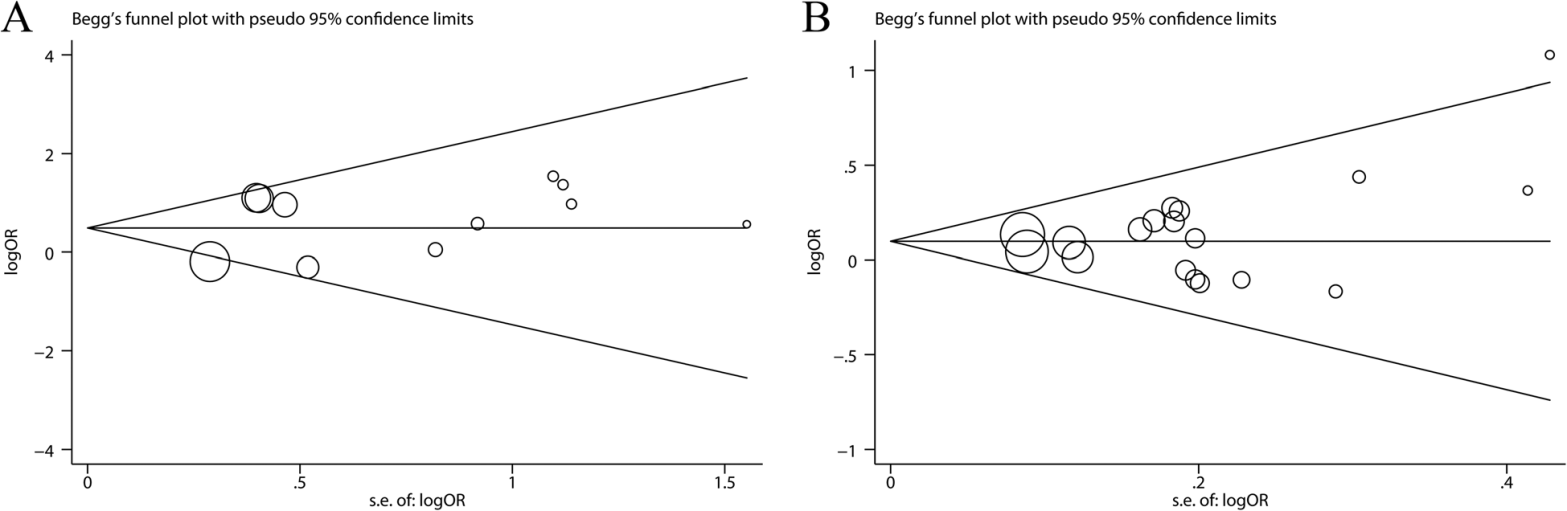
**Funnel plot of two single nucleotide polymorphisms (Arg194Trp and Arg399Gln) in the x-ray repair cross-complementing group 1 gene (*XRCC1*) and bladder cancer risk. A**) Arg194Trp; **B**) Arg399Gln.

There was no evidence of significant publication bias either with the Begg’s test (Figures 3, *P* = 0.640 for Arg194Trp; *P* = 0.820 for Arg399Gln) or with Egger’s test (*P* = 0.345 for Arg194Trp; *P* = 0.248 for Arg399Gln).

## Discussion

The Arg194Trp and Arg399Gln polymorphisms are the most well characterized *XRCC1* polymorphisms, but the reported associations with bladder cancer risk among studies are inconsistent. Our present meta-analysis incorporating 20 case–control studies suggests that the Arg194Trp and Arg399Gln polymorphisms are significantly associated with increased bladder cancer risk.

In this meta-analysis, publication bias was not observed. And there was no obvious heterogeneity between studies. In addition, when repeating the meta-analysis by omitting each study, one at a time, the estimated pooled OR still did not change at all. In view of these findings, we are convinced that the results of our meta-analysis, in essence, are sound and reliable.

The results of the present study are in contrast with a previous meta-analysis published in 2008 [39], which concluded that there was no association between the *XRCC1* polymorphisms and the risk for bladder cancer. However, this study only included ten studies with limited sample size (3,749 cases and 3,947 controls) and, thus, it may lack sufficient statistical power to detect the real association and may have generated a fluctuated risk estimate.

Our findings have some biological plausibility. It is widely accepted that certain genetic variants associated with repair of DNA substantially increase the risk of cancer in carriers because of the alteration of BER functions [40]. BER is the primary DNA damage repair pathway for the repairing of small base lesions resulting from oxidation and alkylation damage [41]. As one of the most important proteins in BER, *XRCC1* is closely related to BER pathway coordination by interacting with most members of the BER short-patch pathway. SNP of *XRCC1* may increase the risk of some types of cancer by damaging the interaction of *XRCC1* with other enzymatic proteins and, consequently, altering DNA repair activity [42]; this may result in carcinogenesis, including a higher incidence of bladder cancer. Similar to the results of our study, *XRCC1* polymorphisms are also reported to be associated with some other cancers. The previous three meta-analyses have confirmed that the Arg399Gln polymorphism is associated with risk of childhood acute lymphoblastic leukemia [43], breast cancer [44], and prostate cancer among Asians [45]. Dai *et al*. reported that the *XRCC1* Arg194Trp polymorphism is associated with an increased lung cancer risk [46] and the study conducted by Li *et al*. suggested that the Arg194Trp polymorphism may be associated with cervical cancer risk [47]. By contrast, in our study, the Arg194Trp polymorphism was associated with disease risk only in Asians, but not in Caucasians. This is mainly because the number of Caucasians is four-fold higher than that of Asians and, therefore, the power to detect association is higher.

Several limitations of this meta-analysis should be mentioned. First, the eligibility criteria for the inclusion of subjects and sources of controls were different from each other. No guarantee could be made among all those eligible studies that there were no potential bladder cancer cases in the controls. Second, because of the lack of the individual original data, our results were just based on unadjusted estimates, and gene–gene and gene–environmental interactions were not addressed in this meta-analysis. Third, although the Begg’s test and Egger’s test did not reveal any evidence of obvious publication bias, some inevitable publication bias may exist, because only studies published in English and Chinese were included in our meta-analysis. Finally, as shown in Table 3, a borderline conclusion (OR: 1.08 (1.00 to 1.18)) of the Arg399Gln section was drawn when two studies without HWE were excluded. This conclusion actually owed much to one study [26] with a relatively large population weight, which implies the need for more well-designed studies in future.

## Conclusions

In conclusion, despite some limitations, the results of our meta-analysis suggest that two polymorphisms in *XRCC1* (Arg194Trp and Arg399Gln) may contribute to bladder cancer development. Whether it could be applied to genotyping for clinical assessment requires large-scale population studies among different ethnicities and regions.

## Abbreviations

BER: base excision repair; CI: confidence interval; HWE: Hardy–Weinberg equilibrium; OR: odds ratio; SNP: single nucleotide polymorphism; XRCC1: X-ray repair cross-complementing group 1.

## Competing interests

The authors declare that they have no competing interests.

## Authors’ contributions

LPX, YQM and XX developed the study concept and participated in its design, data extraction, statistical analysis, manuscript drafting and editing. YWL and HC participated in the literature research, manuscript drafting and editing. JW and ZHH participated in design and data extraction. XLX and YZ participated in manuscript drafting, editing and statistical analysis. All authors read and approved the final manuscript.

## Supplementary Material

Additional file 1: Table S1 Score of quality assessment. Click here for file
