# Supplementary material for: Quantitative assessment of the associations between XRCC1 polymorphisms and bladder cancer risk
Source: World J Surg Oncol. 2013 Mar 7;11:58. doi: 10.1186/1477-7819-11-58 (PMC3601005; doi:10.1186/1477-7819-11-58)
Supplement: Additional file 1: Table S1 — Score of quality assessment. [file 1477-7819-11-58-S1.doc]

| **Table S1. Score of quality assessment** | |
| --- | --- |
| **Criteria** | **Score** |
| Representativeness of case |  |
| Selected from population cancer registry | 2 |
| Selected from hospital | 1 |
| No method of selection described | 0 |
| Representativeness of control |  |
| Population-based | 2 |
| Blood donors | 1.5 |
| Hospital-based (cancer-free patients) | 1 |
| Not described | 0 |
| Ascertainment of bladder cancer |  |
| Histopathologic confirmation | 2 |
| Patient medical record | 1 |
| Not described | 0 |
| Control selection |  |
| Controls matched with cases by age and sex | 2 |
| Controls matched with cases only by age or by sex | 1 |
| Not matched or not descried | 0 |
| Genotyping examination |  |
| Genotyping done blindly  Unblinded or not descried | 1  0 |
| HWE |  |
| HWE in the control group | 1 |
| HWD in the control group or not mentioned | 0 |
| Total sample size |  |
| Larger than 1000 | 3 |
| Larger than 500, but less than 1000 | 2 |
| Larger than 200, but less than 500 | 1 |
| Less than 200 | 0 |
